# A Clinical Lecture on Diseases of Reflected and Sympathetic Nervous Influence

**Published:** 1849-02

**Authors:** Charles A. Lee

**Affiliations:** Prof. of Materia Medica and Pathology


ART. III.—A *Clinical Lecture on Diseases of Reflected and Sympathetic Nervous Influence. Delivered to the Medical Class of the Buffalo University.* By CHARLES A. LEE, M. D., Prof. of Materia Medica and Pathology.

Gentlemen,—I propose in the present lecture, to call your attention to some of the diseases of reflected and sympathetic nervous influence. Dr. Marshall Hall has probably done more to make us acquainted with this class of nervous diseases than all other writers together, and the facts and statements which I shall now make are chiefly the results of his investigations. I have already stated that he has shown that the contractions of all the sphincters, of the œsophagus, the glottis, the iris, the eye lid, and the regular action of the muscles of respiration, are sustained, independently of the will, by a nervous influence, conveyed by *afferent nerves*, from the respective parts or surfaces to the spinal marrow, and reflected from it through the efferent nerves to the muscles connected with these parts. This influence is called the *excito-motory power*. Now, as I have said before, pathology is morbid physiology, and we have but to suppose an increase of this involuntary excito-motory power, and we have disease,—*per se;* as in cases of spasm of the throat and sometimes of the sphincters, in hydrophobia, tetanus, and some hysterical affections. The hurried respiration, the convulsive cough, violent retching, and hiccough, which are occasionally presented in these and other nervous diseases, may also be in part traced to an undue influence of the excito-motory nerves of organic life.

These actions are sometimes excited by sensations, as the respiratory acts, by a feeling of want of breath; cough, by tickling in the air passages; retching by nausea, &c.; but it is where there are no such sensations, or where they bear no proportion to the violence of the actions, that we are warranted to conclude that the excito-motory function is itself exalted. Here you are to remember that at the orifices of passages into the interior, there are peculiar kinds of sensibility connected with the functions of ingestion and egestion; and that these, when modified, constitute a state of disease. Thus the sensation of thirst is but a modification or exaltation of the natural sensibility of the parts, where this sensation originates—and so of craving, nausea, tenesmus, and painful micturition—where the sensibility is impaired, you have anorexia, and paralysis of the rectum and urethra. Another very good example of this exaltation of the excito-motory functions, independent of sensation and volition, is exemplified in the voluntary muscles, when they are deprived of sensation and voluntary motion by disease in the brain itself, or cutting off communication between the brain and spinal cord, without materially injuring the cord itself. Thus in paraplegia from injury of the upper part of the spine, the excito-motory power of the nerves of the lower extremities is exalted, as you will find by tickling, or merely touching the soles of the feet, or legs; convulsive motions immediately are excited, although all voluntary power and sensation are wholly lost. You see the same phenomenon, on cutting off the head of a frog, and touching any part of the surface with a pin, contractions instantly follow—or you may destroy sensation and voluntary motion, in animals or persons, by opium or other narcotics; and then by pinching the skin, you excite spasm, or convulsive actions of the muscles. These experiments prove very conclusively the excited state of the reflex or spinal functions.

In a case of paraplegia, where sensation and motion were entirely lost, I have known the mere touch of the bed-clothes excite troublesome startings. In hemiplegia the same thing occurs, but not often to the same extent; as the cerebral influence is rarely so completely intercepted. Dr. Williams states he has known the convulsive motions of a paralyzed limb so violent in a hemiplegic patient, that it was necessary every night to fasten it down to the bedstead to enable the patient to get sleep.

Wherever you find convulsions, then, according to Marshall Hall, you are to refer them to irritation of the true spinal system. This physiologist has divided the diseases of the spinal marrow into three classes.

I. Central diseases, or diseases of the true spinal marrow itself.

II. Centripetal diseases, or diseases excited through the excitor nerves.

III. Centrifugal diseases, or diseases of the motor nerves.

Of the central diseases, or diseases of the true spinal marrow, itself, we have,

I. Inflammation within the spine—and this may be either of the membranes, or *spinal meningitis*, or

II. Inflammation of the substance of the cord, or *spinal myelitis*—and this may affect,

1st. The cerebral, or sentient and voluntary tracts.

2nd. The true medulla.

3d. The principal divisions.

The *causes* of inflammation within the spine, are various, such as blows, or falls, violent muscular efforts, exposure to damp and cold, intemperance, mental anxiety, intermittent fever, sexual excesses, &c. The worst case I ever saw, was brought on by lifting a heavy piece of timber—another was occasioned by lying on the damp ground.

In cases of meningitis of the spine, you generally find meningitis within the cranium, and it is extremely rare for the membranes of the spinal marrow to be affected, without involving the substance. Hence, these cases are all more or less complicated, making it difficult to define the symptoms peculiar to each. It is fortunate that accurate diagnosis here, is not essential to successful treatment. The locality of the inflammation, according as it affects the medulla oblongata, or the cervical, dorsal, lumbar, and sacral portions of the spinal marrow, is of very great importance. I have known cases, where the whole cord was affected, so that pressing over successive vertebrae, gave rise, first, to dyspnœa; second, to palpitations; third to nausea; a burning sensation in the region of the stomach, and a sense of sinking. In meningitis, the symptoms are more those of irritation of the spinal marrow, or *spasm;* in those of *myelitis,* those of destruction of the organ, or *paralysis.* Both kinds, however, generally co-exist One of the first symptoms ef meningitis, is pain in some part of the spinal column, increased by the motions of the patient, but rarely by pressure over the spine. After a short time, however, the spine becomes tender to the touch, and this symptom will generally serve to distinguish it from myelitis, in which there is usually loss of sensibility. Again, we have spasm in *meningitis.* The head, neck, or trunk, is bent backwards—or there is trismus—wry-neck—opisthotonos, or contraction of the limbs, attended with much pain, or convulsions. Sometimes the respiration is difficult, or there may be constipation, and retention of urine,—the symptoms varying according as the meningitis occupies the upper, middle, or lower part of the spine.

In spinal *myelitis*, or inflammation of the substance of the cord, you find loss of sensation and voluntary motion, a sense of numbness—and feebleness—an impaired muscular power, either in the upper or lower extremities, or in both—There may be, even, augmented sensibility,—also spasms and convulsions—as the disease goes on, the loss of sensation and motion increases—generally the inferior extremities are first paralyzed, then the superior—in some cases, sensation is lost and motion retained—in others motion is lost, and sensation continues.

You will also find that when the disease occupies the upper parts of the spinal marrow, the respiration is impaired, and even the action of the larynx and pharynx becomes affected, and there is difficulty in swallowing, or even asphyxia. I lately attended a fatal case of *myelitis*, which resulted in perfect paraplegia of the lower extremities, with palsy of the bladder, and, where, on dissection, the disease was found opposite the second dorsal vertebra, and in this case, the most prominent symptom, next to a sharp, shooting, burning pain, running through from the spine to the sternum, was the sensation of a cord-like tightness across the epigastrium. When the *lower* part of the spine is affected, the bladder, rectum and their sphincters, are variously paralyzed; and there may be retention of urine, and constipation, or involuntary evacuations, or retention and involuntary flow of urine may be combined. You should always make it a point to ascertain the condition of the bladder and rectum, for as they are unable to evacuate their contents, artificial assistance will be found indispensable. The *morbid anatomy* of these spinal affections, is very similar to that of cerebral meningitis, and myelitis. You find, in spinal meningitis, injection of the pia mater, and of the spinal vessels in general, effusion of serum, lymph, pus, and blood, under the arachnoid, diffused, or in portions—or softening of the adjacent medulla. In *myelitis*, the principal morbid change is softening, which may occupy the whole, or only a portion of the spinal marrow—generally it is confined to the cervical or lumbar portions. When chronic, it is apt to result in induration. The most successful treatment of inflammation within the spine, consists in *cupping* in acute cases, and of *issues* and *setons* in the chronic. Cupping should be applied so as to answer two indications—those of local depletion and counter irritation; and for this purpose, the scarification should be applied deeply and crossed, and little blood should be drawn, the operation being repeated according to the violence of the disease, and the powers of the patient. In chronic cases, issues are indispensable—mercurials, as alteratives are, also, highly useful; the diet should be very mild, and moderate, the bowels kept open, and the patient kept in a recumbent posture and quiet. There is one thing peculiarly noticable in these cases, and that is the state of the mind. In all diseases of the spinal cord, you will find the feelings more or less changed, or perverted. There is great loss of energy and buoyancy of spirit, despondency, melancholly forebodings, great anxiety on the subject of health, apprehension of some severe disease, susceptibility to emotions from slight causes, &c. Instability and fickleness characterize all their thoughts and actions. The bodily symptoms, in addition to those already stated, are disturbances of the digestive organs, as flatulence, costiveness, acidity, affections of the heart and circulation, as manifested in increased or irregular action, or palpitations of this organ; which is an extremely common symptom; derangement of the respiratory organs, as paroxsymal, dry cough, sense of suffocation, convulsive catching inspirations; a sense of sinking, fainting or prostration—accompanied sometimes with giddiness or tinnitus, restlessness or anxiety; in females hysteric paroxysms are a frequent consequence of a morbid condition of the spinal marrow. (In these affections mustard applications, or stimulating liniments, as Granville's lotion to the spine, are often very useful: also mustard pediluvia, and internal diffusible stimulants.) In addition to all these, gentlemen, you will often meet with a variety of anamolous symptoms, or occasional complications, such as a sense of throbing over the whole body—cold extremities—profuse perspiration—chills and rigors, jaundice—affections of the sight and hearing—and derangements of the uterine, urinary, and hepatic systems. All these phenomena are to be taken into consideration, in making up your mind as to the proper treatment.

II. *Congestion* is another form of disease, of the true spinal system; but little is, however, yet known in relation to it, although I have no doubt it is often connected with certain anamolous affections, or convulsive, or hysterical paroxysms, without even being suspected by the physician. Where there is a sudden attack of this kind, without any permanent tenderness over the spine, or particular derangement of any of the important organs, we should be led to suspect congestion of the spinal marrow or its membranes. The treatment is the same as I have pointed out for inflammation of the spinal organs. *Spinal hemorrhage* also belongs to this class of central diseases of the true spinal system. You sometimes hear it called—*spinal apoplexy;* but this is a misnomer—a perversion of language. Apoplexy is the abolition of the functions proper to the *brain*—of sensation, voluntary motion, and thought. The terms—pulmonary apoplexy, hepatic apoplexy, and so on, should therefore never be employed, if we wish to use words in their proper signification. In cases then of spinal hemorrhage, you will have *pain* in some part of the spine; *convulsions; palsy*—the very same symptoms, you perceive, as attend inflammation of the cord and its membranes. It is a rare affection—I have never seen but one case of it in a girl of thirteen, brought on by sudden violent movements of the neck. Dr. Abercrombie has met with only one case of it, and Dr. Bright has never met with it. The symptoms are, sudden pain in the back; spasms; loss of sensation and motion in the lower extremities. The *suddenness* of these symptoms, is the principal circumstance, which will lead to a correct diagnosis.

III. *Centric Convulsions or Epilepsy.*—Marshall Hall remarks, that *any* disease within the cranium or spine, whether effusion, tumor, exostosis, &c, may induce convulsions or epilepsy; while at the same time he maintains that *all* convulsive diseases are diseases of the spinal marrow, and of course that epilepsy is one of these. He brings forward the fact that epileptic convulsions are sometimes excited by *fright* or other mental emotions. This would certainly seem to show that it originates sometimes in the cerebrum, for this is the centre of all the moral emotions and passions, as well as the intellect. We also know that it is occasioned by tumors or exostosis, by which pressure is made upon the brain, or by depression of the bone from external violence—here again the cause acts upon the brain. Now every practitioner is aware that epilepsy is often occasioned by worms, or other sources of irritation seated in the alimentary canal, and one of the worst cases I have ever attended, was owing to the use of alcoholic stimulants. Hence then we have two species at least; one in which the disease originates in the nervous centres themselves, and especially the brain—the other, when it originates in some other part. These two species may be called *centric* and *eccentric*. *Centric*, when the cause lies either in the brain or spinal marrow—the two great nervous centres. *Eccentric*, of course, when the cause is external to these.

IV. Another disease, belonging to the *True Spinal System*, is *Paralysis agitans*. This may be either *general* or *hemiplegic*. It is an affection which if you have once seen, you can never forget. There is a perpetual tremor, even when the part is supported, the head, hands, legs, &c., go incessantly; the patient loses his balance in walking, and is in constant danger of falling forwards; and in order to avoid this he rnns or moves with a quick pace, or on his toes. I once was acquainted with a most severe case of this disease in New York, in a man, who swam across the North river and back, just opposite Hoboken, for a wager; he won it, but he was attacked immediately with this form of paralysis, and has labored under it ever since. This form of disease generally progresses with a steady pace, till it terminates in delirium, lethargy, and death. Another case in which I was lately consulted, was brought on by excessive sexual indulgence.

V. Another disease belonging to this class, is the *Tremor mercurialis*—a paralytic affection, common to workers in mercury, especially those occupied in silvering mirrors. Here we have paralytic tremor, debility, and probably ptyalism; imperfect articulation, imperfect command of the muscles in locomotion, constipated bowels, disturbed sleep, &c.

I. *Centripetal Diseases.*—The first of these noticed by Marshall Hall, is *Centripetal Epilepsy*: that is, when it takes its origin in the *excitor nerves* of the true spinal system, involving the axis of this system, and its motor nerves, in their turn; functionally merely, and not organically. This is the most frequent form of Epilepsy and it is curable; the patient has only to avoid its attack. Its causes are chiefly, 1. The presence of indigestible food in the stomach. 2. The presence of morbid matters in the intestines. 3. Uterine irritation. The first acts through the medium of the pneumo-gastric—the second and third through that of peculiar spinal nerves—all *excitors* belonging to the true spinal system—the *causes* acting through the *excitor* nerves, the *symptoms* being manifested through the *motor* nerves of that system. It does not fall within my province to give a particular description of the symptoms, or treatment of these diseases, but only to throw out such hints, as will aid you in forming a correct diagnosis, as it is not generally difficult to relieve disease, if you know its cause, its nature and its seat. I was recently summoned to visit a boy seven years old, who had fallen from his seat in school, in a fit,—on reaching the place, I found him stretched on his back on the floor,—the eyes wide open and rolled upwards, the pupils extremely dilated,—the right side of the face and body convulsed,—every muscle of that side being in constant convulsive jerks and twitches, while the left side was comparatively unaffected; the pulse was almost imperceptible—breathing exceedingly laborious—froth issued from the mouth—faeces were discharged involuntarily. I opened a vein and let the blood run to the amount of about ten ounces; and while it was running, the spasms became much more violent, but ceased after about one hour had elapsed from the time of attack. He remained about ten minutes free from spasm, when he was again seized, but the convulsive twitches, now, were confined to the left side, while the right remained unaffected. The distortions of the features were most horrible,—every muscle twitched aud jerked, drawing the mouth down to one side,—the flexors of the arm, fore-arm, thigh, leg and feet, were alternately contracted and relaxed, and respiration seemed absolutely suspended; there was the utmost danger that death would speedily result from asphyxia. The face became bloated, and livid, also the hands, and surface generally. This paroxysm lasted about fifteen minutes; when the convulsive actions ceased—consciousness, sensation and volition were not however restored till nearly two hours had elapsed, he lying all this time in a state of stupor; the breathing being stertorous and purely apoplectic. The mouth was filled with viscid saliva, which ran from it in a stream, respiration was much impeded by the quantity of mucus collected in the fauces. While he lay in this state, pinching, pricking, &c., excited no sensation. The cause, in this case, I attributed to worms. A mustard cataplasm to the spine, appeared to be the means of breaking the fit.

II. *Puerperal Convulsion* is also one of the diseases of the excito-motory function—and abortion and parturition, are considered, by Marshall Hall, as phenomena of the same system. The same remark applies to the sickness and vomiting attendant on early pregnancy. The principal causes of puerperal convulsions, besides the peculiar condition of the uterus itself, are indigestible food, a loaded or deranged condition of the bowels, distension of the bladder, mental shock, or anxiety, &c. This form of convulsion closely resembles epilepsy. The treatment varies in different cases, but we must aim at the removal of the causes.

III. *Tetanus* is another disease of the true spinal system. It may be *central* or *centripetal*—central, when it is produced by disease within the spinal canal itself—and centripetal, when it arises from an injured nerve, as in a lacerated, or punctured wound—or it may arise from irritation in the stomach and intestinal canal, as worms, &c. It is a common affection in hot climates, where it is occasioned by sudden changes of temperature and exposure to cold and damp. Infants sometimes are attacked with it, from the condition, it is said, of the umbilicus. In this affection we have what is called the *tonic* spasm, a sort of permanent rigidity, or contraction —on the other hand, *clonic* spasm is what I described as characterizing epilepsy, where the contraction of the muscles takes place repeatedly, forcibly, and in quick succession, the relaxation also being as sudden and frequent. These two kinds of convulsions may be mixed together in the same disease, but it is well to keep the distinction in mind. I have treated several cases of this disease, and I have always found the spasms first effect the muscles about the neck, then those which are concerned in mastication

—when the jaw becomes set, or there is *trismus*—next the muscles of the pharynx, and the deglutition becomes difficult. Next the muscles of the back and of the limbs, are contracted, producing a state of opisthotonos, the cerebral system all the while remaining unaffected. In all the cases of trismus which I have examined after death, and they have been several I have found congestion or inflammation of the cord, and its membranes, and in one instance, an injected state of the nerve, leading from a wound in the foot to the spine. The proper treatment of this affection, is as yet empirical; some recommend cups to the spine, followed by external stimulants, combined with powerful internal stimulants and tonics. The internal use of strychnia has been followed with success in several cases which have occurred in the city of New York, during the last few years, which may be found recorded in the New York Journal of Medicine. Five or six of these were undoubtedly cases of true traumatic tetanus, and they recovered under the use of strychnia; 1-16 of a grain every two hours. I have known of some cases, however, where this treatment has entirely failed.

IV. *Hydrophobia* is another of the *centripetal* diseases of the true spinal system—that is, arising from causes acting at a distance from the nervous centres. This affection arises from the insertion of a poison in the substance of the fine fibrillæ of the excitor nerves—the symptoms manifesting themselves, after a longer or shorter period, depending on circumstances, of which we are entirely ignorant, and all belonging to what Dr. Hall has described as the excito-motory functions. They consist in a peculiar spasmodic and terrible *dysphagia* and *Dyspnœa*,—the parts affected, being those which preside over ingestion. Here, gentlemen, the tri-facial nerves in the face and fauces, and the pneumogastric in the larynx, appear to be most unduly impressible. The impression upon these nerves is reflected upon the muscles of the pharynx and larynx, and the sense of dysphagia, or of dyspnœa is overwhelming. Here, also, the mind often remains clear till towards the last, when delirium sets in, thus proving that like tetanus, it is purely a disease of the true spinal, or excito-motory system.

V. *Hysteria* is another of the centripetal or eccentric diseases of the true spinal system.

This is a truly protiform affection, depending sometimes on the state of the stomach and bowels, sometimes on the condition of the uterine system, and frequently it arises from mental emotions, as grief, terror, anxiety, &c. It is a disease which seems to single out and affect every organ, and every function which belongs to the true spinal system. The respiration, the circulation, the secretions, all are modified by this singular affection. You find the irritation in one case reflected upon the larynx, producing symptoms resembling croup, and apparently threatening suffocation—in another, upon the pharynx, causing dyspnœa, or difficult deglutition—in another, upon the respiratory organs, causing dyspnœa, cough, hiccough, retching and vomiting, &c.,—or upon the bladder, causing dysury, or retention of urine,—or upon the muscular system, giving rise to trismus, tetanus, and spasms, convulsions of every sort—the marvellous phenomena of animal magnetism, are, generally, nothing more nor less, than the morbid manifestations of hysteria.

VI. *Spasmodic Asthma.*—If you attempt to inhale carbonic acid gas, the larynx is instantly closed, through the agency of the pneumogastric or internal excito-motory nerve, as Mr. Hall calls it. The same occurs in the Thymic Asthma, or the crowing inspiration of children, which has sometimes been called laryngeal asthma. In all these cases, the morbid phenomena can only be explained on the principle of *reflex action.* Now suppose you are subject to asthma, and you inhale the powder of Ipecac, it is immediately brought in contact in the bronchi, with the branches of the pneumogastric, or internal excito-motory nerve. The action is reflected by the medulla oblongata, to which the sensation is conveyed, upon the motor branches, and probably upon the circular muscular fibres of the bronchial tubes, which immediately contract, and the phenomena of asthma are produced. Indigestible substances taken into the stomach, and feculent matters retained in the intestines, produce the same effect by their influence upon the incident spinal nerves. Contracted bronchial tubes explain all the phenomena—the dyspnœa—the urgent, rapid, imperfect, bronchial inspiration—the protracted wheezing bronchial expiration—the bronchial rattles under the stethescope, especially during expiration—the secretion of mucus—the cough, &c., &c.

This pathology furnishes a clue to the modus operandi of the inhalation of ether, chloroform, the smoke of tobacco, stramonium and other narcotics. These relax the spasmodic stricture of the bronchial tubes. But how, you ask, does hydrocyanic acid, and other sedatives act when taken into the stomach. They also relieve, by their sedative influence over the true spinal nerves which are morbidly excited; and thus prove remedial. Tonics relieve in spasmodic affections on a different principle—they increase the tonicity of the muscles, and put them in a condition less disposed to take on convulsive action. You are aware that excitability is usually proportioned to the debility of the system—hence, very delicate females, are very impressible—very subject to hysteric convulsions. Raise the tone of the muscles,—increase the general strength, and you relieve this condition, and remove the predisposition to spasm. But to return to *Asthma*; in all cases, the first thing to be done is to remove the exciting cause. If the patient has eaten some indigestible food, and this often brings on an attack, give an emetic; if there is irritation in the intestinal canal, give a cathartic, and aid its operation by a stimulating enema; saturate the atmosphere of the room with moisture, by boiling water over a spirit lamp, or on a stove, if there be one, and apply a fomentation to the chest. By these means, or a dose of morphine, the attack will generally be relieved—that is if the case be one of pure spasmodic asthma. There are asthmatic symptoms depending on lesions of the heart and large vessels, and of organic disease of the lungs, which can only be relieved by relieving the organic changes on which they depend, which of course is out of our power.

VII. *Tenesmus* and *Strangury* are excito-motory phenomena, produced by various irritative causes, as inflammation of the rectum, calculus of the bladder, and even teething in children. They are to be relieved by removing the cause.

VIII. *Sexual Excess or Abuse.*—These are prolific sources of suffering and disease, affecting not only the generative organs, themselves,—but, in many cases, the true spinal system, the cerebral system, in short the whole economy. I know no more prevalent cause of disease than this, whether in male or female, and I have known instances where the practice of self-abuse commenced at two years of age, the child having been taught the practice by its nurse. I have known the health of a little girl of six years, ruined by the practice, which she was taught by an apprentice boy, who lived in the family, and who used to irritate her genital organs with his fingers, as she sat on his lap. These cases, gentlemen, are examples of those that not unfrequently occur in childhood. There is here no instinctive want to gratify—the child is actually trained to the habit by others, till, when the habit is established, like all other habits, it is next to impossible to break it up. Hence the great importance of knowing the moral characters of nurses, and other early companions of children, that their minds be not contaminated with lascivious thoughts, and their constitutions ruined by injurious practices.

A few of the effects of self-pollution are, loss of strength—of flesh, and of digestive power—loss of mental energy, with despondency of the most deplorable kind, and imbecility of mind—extreme debility and emaciation, (tabes dorsalis) phthisis, cachexia, and insanity or idiocy. The reports of our lunatic hospitals, show that large numbers of both sexes are deprived of their rational and moral faculties, by means of this most destructive vice. Excess and abuse of the genital organs induce every kind of derangement of the bladder and kidneys, involving derangement of the stomach, general debility and emaciation, with a host of anomalous symptoms.

All these dreadful results are occasioned by the influence which such abuses exert upon the spinal marrow, deranging by reflex action all the organs and functions, depending on this great nervous centre.

In some of these cases, the whole surface of the body acquires a morbid sensibility, so that the slightest touch of the bed-clothes, will cause a shudder, or even an emprosthotanic spasm, with a slight sob. The remedies for such a state of things are temperance,—a mild but nutritious diet,—active exercise and recreation—regulated thoughts—early hours—salt water shower baths—tepid at first, afterwards cold—cold dash to the loins and genital organs, with moderate cutaneous friction—and iron combined with some vegetable bitter extract internally.

IX. *Abortion* is probably an *excited act* in a generality of cases, and a knowledge of this fact is very important in a prophylactic point of view. You know that it is very apt to be occasioned by a severe attack of dysentery, or cholera; here, irritation is transmitted by the spinal nerves which supply the mucous membrane of the intestinal canal to the spinal marrow; and then contractile efforts are set up by the reflex motor influence which is sent out to the uterus, as well as to other organs. The same thing happens from the irritation caused by the presence of a dead fœtus in the uterus; and in this manner puerperal convulsions are occasioned in such circumstances. One of the most frequent causes of abortion in the early months after marriage, is sexual excesses. I believe nine cases out of ten, happen from this cause—and you know when once occasioned, it is very apt to occur from the operation of similar causes, less in excess, and even from the influence of habit. The grand principle of treatment in these cases, gentlemen, is to avoid impressions upon the *excitor nerves*; uterine contractions—without which abortion can not take place—will not be instituted, unless these nerves are irritated.

You perceive how different is the case with the uterus, and the bladder and rectum. If the former be excited to contractile efforts, its contents are expelled—if the latter, they are obstinately retained. I agree with Dr. Hall that the whole question of abortion and parturition is one of the true spinal system, and we must study it under this aspect if we wish to advance our diagnosis and treatment.

*Incontinence of Urine,* so often met with in young persons, depends on a loaded state of the large intestine, or an acrid condition of the urine, and may be cured by mild aperients, with demulcent enemata, and by the use of diluents containing a small quantity of bicarbonate of potash. Children, troubled with this difficulty, should eat no suppers, and the dinner should be early so that digestion will have been accomplished before bed-time. The best internal remedies are the *pareira brava* in infusion, with from five to ten drops of Tincture of Catharides at bed-time.

III. *Centrifugal Diseases.*—There are certain affections which are diseases of the reflex motor nerves, which Marshall Hall has described under the name of Centrifugal Diseases. Those spasmodic complaints, which originate from causes affecting the *excitor* nerves, and the *spinal* axis, according to this pathologist, I have already enumerated; it now remains briefly to mention those which belong to the latter division.

I. *Spasmodic Strabismus* belongs to this class. You are aware that strabismus may arise from *paralysis* of the cerebral and voluntary nerves, or of some of the muscles of the eye-ball. This variety, which Mr. Hall first pointed out, arises from an affection of the roots of the motor nerves of the true spinal system. In the former kinds of strabismus, the patient can move the eye well enough in every direction but one—where, at a certain point, the eye-ball stops; here the motions of the eye may be perfect, except on certain occasions of excitement, or of disorder, or of intense application, or of employment of the eye; the strabismus then becomes apparent; the eye-ball is evidently drawn in one particular direction. This affection is relieved by dividing the contracted muscle.

II. *Spasmodic Tic.*—Distortions of the face, when permanent, you are aware, arise from paralysis or spasm,—and this may have its seat in the cerebrum, or in the facial nerve. In *hemiplegia*, for example, the eye-lids of the paralytic side are closed by an act of volition, although not so perfectly as those of the unaffected side; the *sensibility* is generally diminished; the *tongue* is protruded *towards* the paralytic side, by means of the contraction of the unaffected side of the genio-hyoid muscle. When the *facial* nerve is paralyzed, the eye-lids can not close at all—the sensibility of the face and the movement of the tongue are natural. Now in the *spasmodic tic*, the countenance is *drawn* to the affected side; and it is the eye of the *same* side, which cannot be closed. This circumstance distinguishes it from *paralysis* of the facial nerve. Sometimes spasmodic tic is attended with defect of vision. Occasionally, the *tic* is confined to the outer portion of the orbicularis muscle. The *causes* of this affection are the usual causes of inflammation; the most frequent, perhaps, is exposure to a keen wind—either extremely cold, or with rain, or sleet. I know of no specific for this disease. The case must be treated on general principles.

III. *Spasmodic Torticollis,* or wry-neck, is nothing but a spasmodic affection of the sterno-cleido-mastoid muscle, and is precisely of the same character as the spasmodic strabismus, and spasmodic tic—an affection of the *true spinal motor* nerves. This is a very common phenomenon after scarlet fever, sometimes becoming permanent, and requiring for its cure a division of the fibres of the muscle. I lately attended a case, brought on by a blow on the side of the head with the fist. Two brothers were at fisticuffs, when the older struck the younger, about eight years old, on the side of the head over the parietal bone, and knocked him down. The head was immediately drawn down towards the right shoulder, the face being turned about a quarter of a circle to the left, and thus remained for two days, when the muscle relaxed, as suddenly as it had contracted, and so remained.

IV. *Spasmodic Respiration* is another of the centrifugal diseases of the true spinal system, which you will find particularly described in Sir Charles Bell's work on the Nervous System.

There are certain diseases of the nervous system of remote origin, which are of great interest and practical importance to the physician and to which I can only allude very briefly. They are—1st, *Intestinal Irritation,* occasioned by indigestible food, scybala, or other morbid contents of the stomach and bowels, excited into activity by some shock of the system, as a fall or other accident, parturition, &c. It is attended with rigor, frequently, severe heat of surface, violent pain of the head, with intolerance of light and sound; in short the symptoms are those of encephalitis. The breath is also very offensive, and the tongue thickly furred, and every thing indicates a morbid state of the secretions,—this will help you very much in forming a correct diagnosis; for these symptoms are not apt to attend simple encephalitis. The best and most effectual way to get rid of scybala, is very copious enemata, passed up with the long tube into the colon, and equal parts of castor oil and turpentine internally as a cathartic in ounce doses for an adult. I have mistaken such cases, gentlemen, for inflammation of the brain, and so have, probably, most physicians; and it has been recommended to apply Marshall Hall's test, of bleeding the patient in an erect position, in order to ascertain the degree of tolerance of blood-letting, and thus be able to determine the nature of the disease. Where the symptoms are occasioned by intestinal irritation, you are aware that the patient will faint from the loss of a very small quantity of blood, while the reverse happens in idiopathic inflammation of the brain.

Gastric irritation is a very frequent cause of convulsions in children. I lately attended a child who was attacked with severe convulsions after being fed with a considerable quantity of cows milk. I got down an emetic, and the child, about two years old, vomited up a piece of solid curd or cheese two inches long and an inch thick. The child had no more spasms, and there can be no doubt that this was the cause of the attack. In another case a small piece of *ham* brought on a severe fit. Andral gives the following case in his Clinic: A middle aged man, 4 days before his entrance into the Hospital, was seized with bilious vomiting, epigastric pain and fever. In 24 hours after the invasion of these symptoms, he first perceived a difficulty in depressing the lower jaw, and a violent trismus was established—which continued for the two following days. At the end of this time he entered the hospital in the following state. Trismus, the head drawn back and forcibly retained in this position by the muscles which are inserted into the occipital region; rigidity of all the extremities; abdomen hard as a board; intellect perfect. Notwithstanding the trismus, the patient could articulate with sufficient distinctness, to give the above account of his case. From the time when the first tetanic symptoms appeared, the vomiting and epigastric pain ceased. He died on the evening of admission. On dissection, no appreciable alteration of structure was found in the brain, or spinal marrow; the meninges of the brain, were very slightly vascular, but those of the spinal marrow pale. The whole surface of the stomach presented an intensely red color. The remainder of the digestive tube was perfectly healthy, and the other organs natural. Here, gentlemen, you have tetanus from gastritis. This is a very instructive case. But you often see spasms, of the most violent kind, in drunkards from the same cause—irritation of the stomach—or rather inflammation, for in most of these cases we have inflammation.

Under this class would also fall—II. *Exhaustion from loss of blood.*—Here you have throbbing pain in the head, or a sense of pressure as if the brain were bound tight with an iron hoop—intolerance of light and sound—sleeplessness—a state bordering on delirium—or even delirium and mania; convulsive affections—perhaps epilepsy itself. In some aggravated cases there will be amaurosis, deafness, paralysis, coma, and even apoplexy. When you find, gentlemen, that this state of things is aggravated, instead of being relieved by blood-letting, it furnishes a very good hint, I think, to stop, and turn about—try a little brandy, ammonia, or animal food, with chalybeates. In this way you will often save your patients, when otherwise they would have died of course. And yet so infatuated are some physicians, that I have actually known cases, where the bleeding has been repeated as many as ten or twelve times within a few days, and each time with an aggravation of all the symptoms, and the patient has at length died, and the physician has said that the patient was unable to bear the treatment necessary to cure him—or the disease was cured, but the patient was too weak to recover —or, some other disease set in and carried him off. So easily do we find excuses to justify ourselves and quiet our consciences, when we are actually guilty of homicide—not intentional, I admit, but scarcely less criminal. At first the symptoms, in exhaustion from loss of blood, are entirely cerebral—next they are partly cerebral, and partly belong to the true spinal system, as the half-closed eye-lid, the convulsions, the stertor, &c. Next the ganglionic system becomes involved, and mucus accumulates in the bronchi, and serum in the air-cells and cellular substance of the lungs; and flatus distends the intestines. After death, you will find effusion under the arachnoid, and at the surface and base of the brain, and into the ventricles; œdema of the lungs, intestines, &c. *Treatment*,—exercise, pure air, nutritious diet, vegetable and mineral tonics, aperients, *&c*. There are other affections connected with this state of the brain, which I have not time to notice more particularly here. These are *Chlorosis, Arthritis, Shock, mental and physical; the Effects of Alcohol, Dropsies, Ischuria, &c.* I may perhaps refer to some of these hereafter.

I have dwelt thus long upon the diseases of the spinal system, because of their great importance, and because their pathology, the offspring of modern science and research, is in the highest degree interesting. You know that these views are very different, from what you find laid down in medical works, and I have no doubt you will agree with me in the opinion that they are far more satisfactory. Thus it is the general doctrine of the books and the schools, that these morbid sympathetic movements or phenomena, are due to the direct nervous connexion, which the great sympathetic nerve establishes between the respective organs; but you perceive that this supposition assumes, what experiment has not proved, that the ganglia of this nerve are either centres of reflection, or sources of nervous influence, which is altogether inconsistent with the latest researches. It is far more probable, and indeed we may consider it established, that the spinal marrow is the centre of reflection in these as in all the other examples of reflex action, which we have been considering, although the sympathetic may be, in many instances, the medium of communication. I advise you, gentlemen, to experiment on this subject for yourselves; you can settle many important principles in your mind in this manner—which without such experiments, would be held as merely loose, hypothetical speculations.

Take a frog or a turtle and decapitate it, then irritate a portion of the exposed spinal cord, and you witness strong contractions of the extremities; now apply this to the human subject; if you see spasms of the limbs, or any other part, will you not, as a matter of course, refer them to irritation within the spinal canal? Or, take the same frog or turtle, and irritate any of the spinal nerves in any part of their course, you have again spasmodic contractions of the muscles of the limb, not only of that side to which the nerve belongs, but also of the other. Here, you perceive, there is no volition, for there is no brain, there can be no sensation in the ordinary meaning of the word—yet you have motion—convulsive motion it is true, but nevertheless motion. Now, what hinders you from applying these physiological facts in explanation of pathological phenomena? Do you not perceive that they throw a broad flood of light upon diseases, which have hitherto been veiled in almost impenetrable mystery? Take the *tonic* spasm of tetanus, you say at once, as the legitimate result of these two experiments, here then I have the true explanation of the pathology of this singular disease. I see this spasmodic condition may be owing either to a diseased state of the spinal marrow itself, or to a morbid condition of the nerves belonging to it. Here is a lacerated wound, or a man has trodden upon a rusty nail, I see that an irritation has been set up at the extremity of certain *incident* nerves. This irritation is conducted along these nerves to the cranio-spinal axis, in which a process or change takes place, whereby an answering influence is reflected to the muscles along the motor nerves; and we have tetanus. But in your experiments on the frog, you found that pinching one of the denuded spinal nerves, not only set both hind legs in motion, but also the fore legs, thus exciting spasmodic actions *above* as well as *below* the junction of that nerve with the cord. You then infer, for you see the fact demonstrated before your eyes, that the change, whatever it be, that is wrought in the cord by impressions made on one of its afferent nerves, is not necessarily confined to the corresponding *segment* of the cord; but is communicated in both directions through its entire course; the whole of this centre of the excito-motory system responding to the influence conveyed by a single nerve, as completely as a tight string vibrates from end to end, when struck at any one point.

This pathology furnishes us with a satisfactory explanation of some sympathetic sensations, which, otherwise, would remain entirely inexplicable.

How else can we account for impressions made on the ultimate distribution of one nerve producing sensations in parts supplied by another nerve, or by another branch of the same nerve—as when you cause a tickling sensation in the glottis by touching the external auditory meatus. On this principle only can we account for the well known fact that a calculus in the bladder produces pain at the extremity of the penis; or ascarides in the rectum, itching of the anus, or pudendum; or congestion of the liver, pain at the top of the right shoulder; or a disordered state of the stomach, pain in the left shoulder-blade. Why, in angina pectoris, do we have pain extending *down* the arms; or in painter's colic, pain in the legs and feet; or in gastric derangement, headache, &c? In these and other instances, that might be mentioned, the sensations can not be referred to direct nervous communication, but to an influence reflected from the spinal centre only.

I shall here leave this subject, which is full of the highest interest to the pathologist, with the hope that what I have thrown out—and I am aware that they are merely hints—will be the means of calling your attention to it hereafter, when you are engaged in the practice of your profession.

